# A pilot randomized controlled trial of WeiNaoKang (SaiLuoTong) in treating vascular dementia

**DOI:** 10.1002/agm2.12230

**Published:** 2022-11-22

**Authors:** Junguang Liu, Dennis Chang, Dennis Cordato, Kien Lee, Hugh Dixson, Alan Bensoussan, Daniel Kam Yin Chan

**Affiliations:** ^1^ NICM Health Research Institute Western Sydney University Westmead New South Wales Australia; ^2^ Department of Neurology Liverpool Hospital Liverpool New South Wales Australia; ^3^ Department of Nuclear Medicine and Ultrasound Bankstown‐Lidcombe Hospital Bankstown New South Wales Australia; ^4^ University of New South Wales Kensington New South Wales Australia; ^5^ Department of Aged Care & Rehabilitation Bankstown‐Lidcombe Hospital Bankstown New South Wales Australia

**Keywords:** cerebral perfusion scan, dementia, randomized controlled trial, SaiLuoTong, vascular dementia, WeiNaoKang

## Abstract

**Objective:**

WeiNaoKang (or SaiLuoTong) is an herbal formula consisting of ginkgo, ginseng, and saffron. Our objective was to investigate if WeiNaoKang could improve cognitive function and cerebral perfusion in patients suffering from vascular dementia.

**Methods:**

A 16‐week randomized double‐blind, placebo‐controlled trial was carried out in the setting of a memory disorder clinic at a single center. Patients with vascular dementia diagnosed clinically but supported by imaging and other investigations were invited to participate. The diagnoses were based on the National Institute of Neurological Disorders and Stroke/Association Internationale pour la Recherche et l'Enseignement en Neurosciences (NINDS‐AIREN) criteria. An independent blinded assessor evaluated the effects of the formula. Intervention group was compared to the control group. A subgroup of participants was randomly chosen for further evaluation of cerebral perfusion by single photon emission computed tomography scans post‐treatment.

**Results:**

Both groups were comparable in age (mean = 74 ± 7.2 years in the placebo group and 75 ± 7.4 in the intervention group) and in other demographics. Sixty‐two participants were included in final analysis. Alzheimer's Disease Assessment Scale – Cognitive Portion (ADAS‐cog) was the primary outcome. By week 16, the mean ADAS‐cog reduced from 24.48 to 20.30 (mean reduction = 4.18) for those in the treatment group, and from 18.98 to 17.81 (mean reduction = 1.18) for those in the placebo group. The difference in mean reduction of ADAS‐cog was −3.00 (95% confidence interval [CI] = −4.910 to −1.100) in favor of the treatment group. Secondary outcomes of activities of daily living and quality of life measures also showed significant difference. In the perfusion scan analysis, the difference in the change in cerebral blood flow (t‐scores) pre‐ and post‐treatment between the intervention group (n = 7) and the placebo group (n = 11) was statistically significant (*P* < 0.001).

**Conclusion:**

In this randomized, double‐blind placebo‐controlled trial, we demonstrated significant differences in improvement in cognitive function and activities of daily living. The clinical improvement is corroborated with improvement in cerebral perfusion in a subset of participants.

## Graphical abstract

A 16‐week randomized double‐blind, placebo‐controlled trial for vascular dementia was carried out with WeiNaoKang (or SaiLuoTong). There were significant differences in improvement in cognitive function and activities of daily living, favouring the treatment group. The clinical improvement is corroborated with improvement in cerebral perfusion in a subset of participants. Difference in the changes of cerebral blood flow (t‐scores) pre‐ and post‐treatment between the treatment (WNK) group and the placebo group for patients with vascular dementia is shown in the SPECT image. The red colour represents the increases in cerebral blood flow.

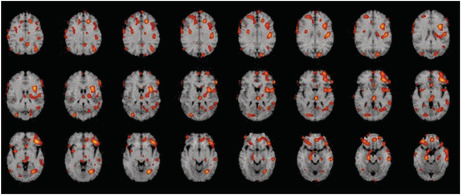

## 1 INTRODUCTION

### 1.1 Background

According to the World Health Organization, there are currently more than 55 million people worldwide who live with dementia, and there are nearly 10 million new cases every year.^1^ Vascular dementia (VaD) is generally regarded as the second most common cause of dementia, after Alzheimer's disease (AD), accounting for about 15% of all dementia cases,^2^ although the estimate is higher in Asia, constituting around 30% of cases and higher still in East Asia.^3^, ^4^ Unlike AD, to date, there is no licensed treatment for VaD. Therefore, an intervention that is inexpensive and easily accessible will be of great promise for persons with VaD.

WeiNaoKang (WNK), currently known as SaiLuoTong (SLT), is a novel standardized herbal formula that consists of three commonly used Chinese herbs for treating dementia‐like symptoms. It has shown promising results and a recent SLT randomized controlled trial (RCT) has found at week 26, the improvement in the VaD Assessment Scale–cognitive subscale scores was 2.67 (95% confidence interval [CI] = 1.54–3.81) for group A (higher SLT daily dose, 360 mg) vs controls, and 2.48 (1.34–3.62) for group B (lower SLT daily dose, 240 mg) vs controls (both *P* < 0.001).^5^ This is only one of the few herbal medication treatments for VaD that is published in an international journal outside of China. Although this novel finding is interesting, further supportive evidence, such as imaging in the improvement of cerebral perfusion in the treatment group, would be very useful in building a stronger case for the generalizable use of this combination herb.

Prior to the aforementioned published paper, we had conducted a pilot RCT using WNK at Bankstown‐Lidcombe Hospital, Sydney, Australia, and for a subset of patients (both treatment group and controls), single photon emission computed tomography (SPECT), a perfusion nuclear imaging investigation was applied. We hereby report our findings in this paper of the clinical outcomes and imaging findings as further evidence for the use of WNK in the treatment of VaD.

### 1.2 WeiNaoKang (or SaiLuoTong)

WNK or SLT is a complex herbal formulation that consists of standardized extracts of *Ginkgo biloba* (Ginkgo), *Panax ginseng* (Ginseng), and *Crocus sativa* (Saffron). In searching the English literature, the aforementioned article by Jia et al^5^ is the only clinical study of this herbal combination published for the treatment of VaD. We have reviewed the evidence of individual herb (ginkgo, ginseng, and saffron) and summarize as follows.

#### 1.2.1 Ginkgo biloba

The main active components of ginkgo biloba include quercetin, kaempferol, ginkgolides (eg, ginkgolide B and ginkgolide C), and bilobalide.^6^ The results of previous research suggest that ginkgo biloba possesses the following pharmacological effects (i) antioxidant, (ii) anti‐inflammatory, (iii) increase glucose uptake and ATP production, (iv) improve blood flow by inducing nitric oxide production, and (v) inhibition of platelet activating factors.^6^

One recent review and meta‐analysis of ginkgo biloba that included treatment of VaD and AD, identified nine good quality trials using the standardized extract EGb761.^7^ Trials were of 12 to 52 weeks duration and included 2372 patients in total. In the meta‐analysis, the standardized mean differences (SMDs) in change scores for cognition were in favor of ginkgo compared to placebo (−0.58, 95% CI = −1.140 to −0.010, *P* = 0.040), but did not show a statistically significant difference from placebo for activities of daily living (ADL; SMD = −0.32, 95% CI = −0.660 to 0.030, *P* = 0.08). Heterogeneity among studies was high. For the AD subgroup, the SMDs for ADL and cognition outcomes were larger than for the whole group of patients with dementias with statistical superiority for ginkgo also for ADL outcomes (SMD = −0.44, 95% CI = −0.770 to −0.120, *P* = 0.008).^8^ Dropout rates and side effects did not differ between ginkgo and placebo. No consistent results were available for quality of life and neuropsychiatric symptoms, possibly due to the heterogeneity of the study populations. The authors concluded that ginkgo biloba EGb761 at the dose of 240 mg/day appears more effective than placebo. However, effect sizes were only moderate. The conclusion was also echoed in a subsequent systematic review by Hong‐Feng Zhang et al.^9^

More recently, a RCT of ginkgo biloba extract,^10^ a RCT of Shemayishi formula in combination with ginkgo extract tablets,^11^ and a retrospective analysis of ginkgo biloba extract EGb 761 with or without cholinesterase inhibitors^12^ have demonstrated improvement in vascular endothelial function and slowing down or improvement of cognitive impairment in subjects with VaD.

#### 1.2.2 Panax ginseng

Ginseng, the root of Panax ginseng C. A. Mey is a widely used herb to treat VaD. The principal bioactive components of ginseng are ginsenosides (eg, ginsenosides Rg1, Rg3, and Rg5), which have been suggested to have antioxidant, anti‐inflammatory, and anti‐apoptotic effects. In addition, ginsenoside Rg5 has been shown to reduce amyloid and cholinesterase activity, whereas ginsenoside Rg3 has also been shown to promote amyloid peptide degradation via enhancing gene expression. In addition, research demonstrates that Panax ginseng decreases blood pressure and improves blood circulation via vasodilation activities.^6^

An older review^13^ on the effect of ginseng on cognitive function of healthy individuals found five published studies eligible for analysis. However, the authors concluded that there was a lack of convincing evidence to show a cognitive enhancing effect of Panax ginseng in healthy participants and no high‐quality evidence about its efficacy in patients with dementia.

In a more recent review,^6^ the authors found clinical trial data suggesting that ginseng modestly improved thinking and working memory in healthy volunteers.^14^, ^15^ Two open‐label trials showed that 12‐week treatment with ginseng improved AD Assessment Scale‐Cognitive Subscale (ADAS‐cog) scores in participants with AD.^16^, ^17^

Two small open‐label trials demonstrated the potential therapeutic benefits of Panax ginseng for AD.^17^, ^18^ In the former study, which showed significant effects on ADAS‐cog and Clinical Dementia Rating (CDR) following 24‐week treatment of low or high dose (4.5 g or 9 g/day) Panax ginseng compared with controls,^16^ subjects were followed up for a further 2 years during which time cognitive function was evaluated every 12 weeks using the ADAS and the Korean version of the Mini‐Mental State Examination (K‐MMSE). In the long‐term efficacy evaluation of the effect of Panax ginseng, cognitive function was sustained for the follow‐up period. In the latter study, in which 97 participants with AD (58 in the ginseng group and 39 in the control group) were involved, 12‐week treatment with Panax ginseng powder (4.5 g/day) produced significant improvements in ADAS‐cog and MMSE scores.^17^ Clinical benefits have also been demonstrated after Panax ginseng is combined with ginkgo in improving cognitive function in healthy subjects.^6^

However, we could not find any specific review or original articles for treatment of VaD with ginseng. Furthermore, most of the studies mentioned before in treating AD were small in sample size, short in duration (maximal 24 weeks) or were open labeled trials, making it difficult to draw strong conclusions due to the possibility of bias.

#### 1.2.3 Saffron

Saffron (Crocus sativus or Xi Hong Hua) has multiple active ingredients, including carotenoids, flavonoids, terpenoids, amino acids, and alkaloids.^19^ Saffron has reported anti‐inflammatory, anti‐platelet, and anti‐apoptotic properties.^6^

In a recent review^20^ that covered both English and Chinese literature, a total of four RCTs were included. The analysis revealed that saffron significantly improves cognitive function as measured by the ADAS‐cog subscale and Clinical Dementia Rating Scale‐Sums of Boxes (CDR‐SB), compared with the placebo groups. There was no significant difference between saffron and conventional medicine, as measured by cognitive scales, including ADAS‐cog and CDR‐SB. Saffron improved daily living function, but the changes were not statistically significant. Adverse events were mild. The author concluded in saying that saffron has the potential to improve cognitive function and ADL, but the study was limited by its small sample size and has not been published as a full article.

### 1.3 Aims

Our primary aim was to evaluate the efficacy and safety of WNK on cognitive function and secondary aims were to (i) investigate effect of WNK on quality of life and ADL in patients with VaD and (ii) investigate the effect of WNK on the brain blood flow (using SPECT) in patients with VaD.

## 2 METHODS

### 2.1 Study design and participants

This study was a 16‐week randomized, double‐blind, placebo‐controlled clinical trial. The trial was conducted in full compliance with the International Conference on Harmonization (ICH) guidelines on Good Clinical Practice (GCP) and the Australian Therapeutic Goods Administration (TGA) guidelines for clinical trials. It was approved by both the Western Sydney University Human Research Ethics Committee (approval no: 04/061) and the Sydney South Western Area Health Service Human Ethics Committee (approval no: 04/057).

### 2.2 Setting

Potential participants were recruited from the Aged Care Outpatient Clinics and the Memory Disorder Clinics at Bankstown‐Lidcombe Hospital.

### 2.3 Inclusion and exclusion criteria

#### 2.3.1 Inclusion criteria

Participants were required to be age 60 years old and over, with absence of severe depression (as screened for by the Geriatric Depression Rating Scale 15‐item version, total score < 11). Patients with a history of mild depression were permitted to join the trial if they were stable on antidepressant medication for over 3 months. Patients on cholinesterase inhibitors were also allowed to participate if there had been no significant clinical change over the last 3 months with the medication as evidenced by unchanged ADAS‐cog and MMSE scores. Patients who had comorbidities, such as hypertension, diabetes, cardiac disease, or stroke, were included if these disorders were stable or controlled by medication for at least 3 months.

#### 2.3.2 Exclusion criteria

Patients with other types of dementia, delirium, schizophrenia, acute illness, significant liver or renal disease, or other poorly controlled chronic diseases were excluded. Patients who were already on products containing any ingredient of WNK/SLT and those taking psychotropic drugs or hypnosedatives were excluded, as were patients on drugs, such as warfarin, that could have significant drug interactions with the herbs in the formula.

### 2.4 Cases ascertainment

All cases were assessed by geriatricians and/or neurologists who were trained and had specialized skills in assessing memory disorders. All participants underwent a thorough history taking, physical examination, cognitive testing (MMSE and ADAS‐cog), Geriatric Depression Scale, and daily living assessment scales. In diagnostically difficult cases, a neuropsychologist was available for assistance.

All participants had blood tests to examine any reversible factors (B_12_, folate, thyroid function, and syphilis screen), as well as renal and liver functions. They also had either a computed tomography (CT) scan or magnetic resonance imaging (MRI) scan of the brain. In addition, at baseline, all had a ^99m^Tc‐HMPAO SPECT cerebral perfusion scan.

The SPECT images were processed on a Siemens ICON workstation (Siemens Medical Systems) using filtered back‐projection and attenuation corrected using Chang's Method. The reconstructed images were aligned on the occipitofrontal line and displayed using MedView software (MedImage). Images were also normalized, mapped onto the Talairach stereotactic brain space, compared to normal, age‐specific databases, and overlayed onto a standard brain MRI using the Neurostat software package (Neuroimaging and Biotechnology Group, Department of Radiology and Imaging Sciences, University of Utah).

### 2.5 Diagnostic criteria

The diagnoses of probable or possible VaD, or mixed VaD and AD of more than or equal to 3 months duration were based on the National Institute of Neurological Disorders and Stroke/Association Internationale pour la Recherche et l'Enseignement en Neurosciences (NINDS‐AIREN) criteria (Table 1).

**TABLE 1:** The NINDS‐AIREN criteria for patient inclusion.

I. Dementia (1 and/or 2)
1. Cognitive decline
Impairment of memory Impairment of cognitive domains (two or more) Orientation Attention Language Visuospatial functions Executive functions Motor control Praxis
2. Clinical feature consistent with probable vascular dementia (one or more needed)
Early presence of gait disturbance History of unsteadiness and frequent, unprovoked falls Early urinary frequency, urgency, and other urinary symptoms not explained by urologic disease Pseudobulbar palsy Personality and mood changes, abulia, depression, emotional incontinence, or other subcortical deficits including psychomotor retardation and abnormal executive function
II. Cerebrovascular disease (1 and 2)
1. The presence of focal signs on neurologic examination, such as: (one or more needed)
Hemiparesis Lower facial weakness Babinski sign Sensory deficit Hemianopia Dysarthria consistent with stroke
2. Evidence of relevant cerebrovascular disease by imaging (CT or MRI) including: (one or more needed)
Multiple large vessel infarcts, or A single strategically placed infarct, or Multiple basal ganglia, or White matter lacunes, or Extensive periventricular white matter lesions Combinations
III. A relationship between the above two disorders: (1 or 2)
1. Onset of dementia within 3 months following a recognized stroke
2. Abrupt deterioration in cognitive functions; or fluctuating, stepwise progression of cognitive deficits

Abbreviations: CT, computed tomography; MRI, magnetic resonance imaging; NINDS‐AIREN, National Institute of Neurological Disorders and Stroke/Association Internationale pour la Recherche et l'Enseignement en Neurosciences.

### 2.6 Randomization and blinding

Using a computer randomization generator, a statistician generated balanced randomization with no access to information on the patients and investigator. Blocking was used to ensure a close balance of the numbers in each group. Participants and investigating staff were blinded to treatment allocation until the end of the trial. Independent pharmacists from the Bankstown Hospital Pharmacy Department dispensed either WNK or placebo according to the computer‐generated randomization list.

All participants were consented before randomization.

### 2.7 Sample size and power calculations

We estimated that for adequate power (80%) to detect a 20% difference on the ADAS‐cog scores at the 5% significant level (two‐tailed test), at least 30 patients were needed in each group. Allowing for 20% dropouts in the active arm, with full compliance in the placebo arm, 70 subjects were recruited, namely, 35 subjects in each arm.

### 2.8 Study intervention

#### 2.8.1 Chemical and safety profiles of the herbs

All herbs used in this trial are listed with the Federal Government's Therapeutic Goods Administration and have such been acknowledged as suitable for human consumption. All herbs are currently available over the counter to the public throughout Australia and are classified as food products or herbal ingredients.

#### 2.8.2 WNK and placebo preparation

The Research Centre, Xiyuan Hospital, China Academy of Chinese Medical Sciences, developed the formula for WNK and the placebo. Tianjin Zhongxin Pharmaceutical Group Co. Ltd., a pharmaceutical manufacturer in China with an Australian Good Manufacturing Practice license, prepared the trial medications. The placebo was prepared and designed to taste, smell, and look the same as the Chinese herb medicine (CHM) formulation.

WNK and the placebo were in the form of capsules pre‐packaged in blister packs each containing 60 mg of the active ingredients (ratios of the ingredients by weight being 5:5:1 with the least amount being saffron). The dosage of the WNK in the clinical trial was one capsule, three times daily by oral administration.

### 2.9 Data collection and analysis of efficacy parameters

Data were collected at various stages: baseline, at 8 weeks, and 16 weeks, during the course of the trial and at 32 weeks (or 16 weeks after trial completion). An SPSS database was established to collect and keep data, in keeping with GCP guidelines. The Last Observation Carried Forward (LOCF) method was used for the missing observations. Analysis of both observed cases at each scheduled visit and LOCF at week 16 was conducted. Week 16 LOCF using the ADAS‐cog was defined as the primary end point evaluation for each patient. Analysis of safety was performed on the population that included all patients who received at least one dose of study medication and who provided any post‐baseline follow‐up data.

An independent research assistant (blinded to group allocation) was engaged to calculate all the scores and data entry. All data analysis was carried out using SPSS 14.0 software according to a pre‐established analysis plan. Pearson product moment correlation and Cronbach alphas were used in the analysis of reliability and validity data, and factor analysis was used to determine construct validity. Independent Sample *T* Test, one‐way analysis of variance (ANOVA), or analysis of covariance (ANCOVA; when necessary) was used to determine the differences among groups at baseline, end of treatment, and follow‐up. Outcome measures with categorical responses were analyzed using the chi‐squared or Fisher's exact tests (when necessary). The main outcome included the odds ratio and 95% CIs. All tests would be two‐tailed and conducted at the 5% significance level. Data involved all patients who were randomly assigned. All data were monitored and double checked for anomalies and consistency by a data entry assistant blinded to the purpose of the study.

### 2.10 Outcome measures

ADAS‐cog was used as a primary measure to assess the cognitive function.

Secondary outcomes measured included MMSE, Alzheimer's Disease Cooperative Study ‐ Activities of Daily Living (ADCS‐ADL) and change in the scores of Short Form **(**SF‐36) Health Survey assessed by the patients and the investigators.

A subset of 18 subjects were randomly chosen for repeated SPECT scans at the baseline and end of the trial. The primary outcome measure of the SPECT study was changes in cerebral blood flow (CBF) in various brain regions between the WNK group and the placebo group at the end of treatment. Pre‐ and post‐intervention in both the treatment group and the placebo group were also assessed. The normalized, registered pre‐ and post‐treatment images were compared within each of the treatment and control groups and a t‐score of the change computed for each point within the brain for each group. Finally, the t‐score changes were compared between groups. This is a standard approach in the analysis of SPECT neuroimaging.

## 3 RESULTS

### 3.1 Demographic data and baseline characteristics of participants

**FIGURE 1:**
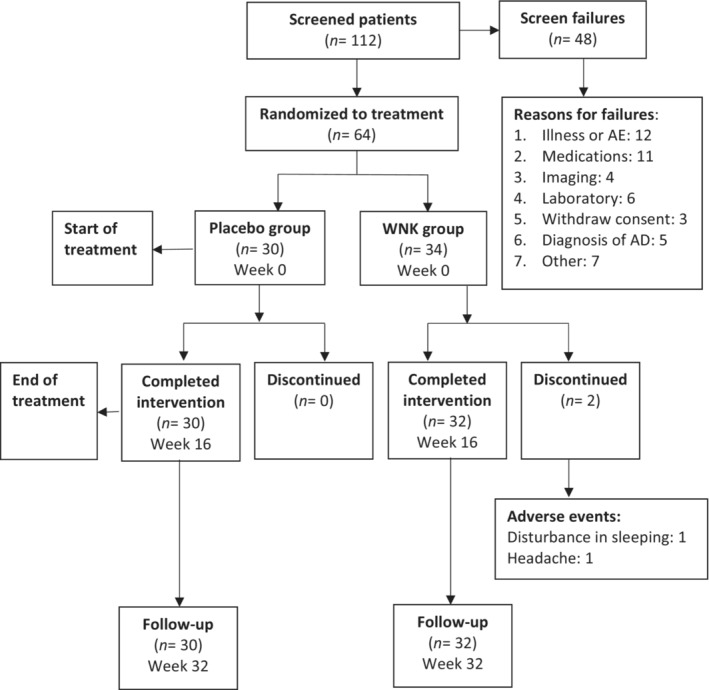
Patients’ progress through stages of the trial. AD, Alzheimer's disease.

Participant data on study entry are shown in Table 2. Participant groups were similar in terms of age (mean = 74.1 ± 7.2 in the placebo group and 75.0 ± 7.4 in the WNK group), sex distributions, language spoken at home, and education level. One hundred twelve persons were screened, with 48 subjects excluded (Figure 1). Sixty‐four participants were recruited but two withdrew from the study and were not included in the final result for analysis according to our study protocol. Among the 62 participants included for final analysis, 22 were men, and 40 were women. There were 49 participants with probable VaD and 13 participants with possible VaD, based on the NINDS‐AIREN criteria. The ratios of probable VaD vs possible VaD in placebo and WNK group were 25:5 and 24:8, respectively. There was no statistical significance between the two groups (*P* = 0.42). In addition, no significant differences were found between patients in the two groups in terms of total severity of symptoms as judged by the MMSE (*P* = 0.47). The ratios of mild vs moderate VaD in the placebo and the WNK groups were 14:16 and 12:20, respectively.

**TABLE 2:** Participants’ characteristics at baseline.

Characteristic	Placebo group (n = 30)	WNK group (n = 32)	*P* value
Diagnosis			
Probable vs possible VaD	25:5	24:8	0.42
Mild vs moderate VaD	14:16	12:20	0.47
Language spoken at home (English vs no English)	21:9	20:12	0.53
Mean education level ± SD, y	9.6 ± 3.5	9.3 ± 3.7	0.79
Mean age ± SD, y	74.1 **±** 7.2	75.0 **±** 7.4	0.59
Sex ratio (male: female)	10:20	12:20	0.73
Family history of dementia/memory			
Impairment, n (%)	7 (23.3%)	12 (37.5%)	0.38
Co‐existing diseases, n (%)			
Hypertension	15 (50.0%)	16 (50.0%)	1.00
Clinical identified stroke/TIA	8 (26.7%)	10 (31.3%)	0.77
High cholesterol	6 (20.0%)	8 (25.0%)	0.71
Diabetes	5 (16.7%)	4 (12.5%)	0.69
Heart disease	11 (36.7%)	7 (21.9%)	0.34

*Note*: Nonsignificant *P* values are expressed to two decimal points only in all tables.

Abbreviations: TIA, transient ischemic attack; VaD, Vascular dementia; WNK, WeiNaoKang.

### 3.2 Alzheimer's Disease Assessment Scale – Cognitive Portion

For the primary outcome measure, ADAS‐cog as assessed by investigators, demonstrated that patients receiving the WNK formulation responded significantly better than patients receiving placebo.

#### 3.2.1 Week 8 assessment

At week 8 of treatment, the means of ADAS‐cog in both the WNK and placebo groups were reduced. By week 8, the mean ADAS‐cog decreased from 24.48 to 22.47 (mean reduction = 2.01) for those in the WNK group, and from 18.98 to 17.39 (mean reduction = 1.59) for those in the placebo group (Table 3). The difference in mean reduction between the WNK group and the placebo group in ADAS‐cog were −0.43 (95% CI = −1.790 to 2.640; *P* = 0.70).

**TABLE 3:** Changes of participants’ mean total ADAS‐cog scores at baseline and week 8 of treatment (mean ± SE).

Group	n	Mean difference between baseline and week 8	Mean difference between groups	95% CI lower and upper		*P* value (two tailed)
Placebo	30	−1.59 ± 0.79	0.43 ± 1.11	−1.790	2.640	0.70
WNK	32	−2.01 ± 0.78				

Abbreviations: ADAS‐cog, Alzheimer's Disease Assessment Scale – Cognitive Portion; CI, confidence interval; WNK, WeiNaoKang.

#### 3.2.2 Week 16 assessment

After week 8, the scores of ADAS‐cog in the WNK group continued to decline until the end of treatment, whereas that in the placebo group slightly increased. The mean reduction in ADAS‐cog from the baseline to week 16 was significantly greater for those receiving WNK than those receiving placebo. By week 16, the mean ADAS‐cog went from 24.48 to 20.30 (mean reduction = 4.18) for those in the active treatment with WNK and from 18.98 to 17.81 (mean reduction = 1.18) for those in the placebo group. The difference in mean reduction between the WNK treated group and the placebo group in ADAS‐cog was −3.00 (95% CI = −4.910 to −1.100; Table 4).

**TABLE 4:** Difference of participants’ mean total ADAS‐cog score at start and end of treatment (mean ± SE).

Group	*n*	Mean difference between baseline and end of treatment	Mean difference between groups	95% CI	*P* value (2 tailed)
Placebo	30	−1.18 ± 0.58	3.00 ± 0.95	1.100 to 4.910	0.003
WNK	32	−4.18 ± 0.75			

Abbreviations: ADAS‐cog, Alzheimer's Disease Assessment Scale – Cognitive Portion; WNK, WeiNaoKang.

It was noted that there was a baseline difference between the two groups, although the difference was not judged to be clinically significant. To test whether WNK still had an effect on ADAS‐cog score after removing the baseline differences, the ANCOVA was performed with the baseline ADAS‐cog score as a covariate. The ANCOVA also showed that the mean difference between groups was statistically significant (F [df1, df2] = 9.000, *P* = 0.004).

#### 3.2.3 Week 32 (that is 16 weeks after completion of the 16‐week study)

Sixteen weeks after the completion of the treatment, the assessment of ADAS‐cog was conducted to observe any residue effects of the intervention. Results showed that the treatment effects discontinued and the patients’ symptoms in both groups were slightly worse. The mean total of ADAS‐cog increased from 20.30 to 21.92 (mean increase = 1.61 ± 0.75) for those in the WNK group, and from 17.80 to 19.52 (mean increase = 1.71 ± 0.81) in the placebo group. The difference in mean increase between the WNK group and the placebo group in ADAS‐cog were − 0.1 (95% CI = −2.110 to 2.310; *P* = 0.93). There was no significant difference between the WNK and the placebo groups (Table 5).

**TABLE 5:** The changes of participants’ mean total ADAS‐cog score at week 16 and follow‐up of treatment (mean ± SE)

Group	N	Mean difference between week 16 and week 32	Mean difference between groups	95% CI	*P* value (two tailed)
Placebo	30	1.71 ± 0.81	−0.1 ± 1.10	−2.110 to 2.310	0.93
WNK	32	1.61 ± 0.75			

Abbreviations: ADAS‐cog, Alzheimer's Disease Assessment Scale – Cognitive Portion; CI, confidence interval; WNK, WeiNaoKang.

### 3.3 Mini‐Mental State Examination

The MMSE scores of patients improved after the 16 week treatment in the WNK group. The mean differences between the baseline and the end of treatment in the WNK group and the placebo group were 1.2 and 0.3, respectively (Table 6). The inter group difference was 0.92 (95% CI = −1.880 to 0.450), which just failed to reach statistical significance (*P* = 0.06).

**TABLE 6:** Changes of participants’ mean total of MMSE at start and end of treatment (mean ± SE)

Group	n	Mean difference between baseline and end of treatment	Mean difference between groups	95% CI	*P* value (two tailed)
Placebo	30	0.30 ± 0.29	−0.92 ± 0.48	−1.880 to 0.450	0.06
WNK	32	1.22 ± 0.37			

Abbreviations: CI, confidence interval; MMSE, Mini‐Mental State Examination; WNK, WeiNaoKang.

### 3.4 ADL results

Total mean of ADCS‐ADL independent‐sample *t* test was used to determine the mean differences of ADL between treatment groups. Although the mean total scores from baseline to week 16 in the WNK group increased 2.13, compared with 0.73 in the placebo group, the mean difference had no statistical significance (*P* = 0.24). However, 63% of the patients in the WNK formulation group improved their ADL as assessed by carers using ADCS‐ADL, compared with 33% of the patients in the placebo group (*X* ^*2*^ = 7.224; *P* = 0.027; Fisher's exact test; *P* = 0.026; Table 7).

**TABLE 7:** Improvement by treatment group – ADL compared with before trial, n (%)

	WNK group	Placebo group	*P* value
Improved	20 (62.5%)	10 (33.3%)	
Stayed the same	6 (18.8%)	5 (16.7%)	
Worsened	6 (18.8%)	15 (50.0%)	0.026

Abbreviations: ADL, activities of daily living; WNK, WeiNaoKang.

### 3.5 Short Form Health Survey‐36 results

Item 2 in the SF‐36 Health Survey is a general health question which asks participants “Compared to one year ago, how would you rate your health in general now?” Although there were no significant differences for the SF‐36 Item 2 between the WNK and the placebo treatment groups on commencement of the treatment, participants in the active group reported significant improvement over those in the placebo group by the end of the treatment (Table 8).

**TABLE 8:** SF‐36 Item 2 Difference in overall health improvement reported by WNK and placebo groups at end of treatment (Chi‐squared), n (%)

	WNK group	Placebo group	*P* value
Much or somewhat better	18 (56.3%)	6 (20.0%)	
About the same	10 (31.3%)	12 (40.0%)	
Much or somewhat worse	4 (12.5%)	12 (40.0%)	0.006

Abbreviations: SF‐36, Short Form Health Survey‐36; WNK, WeiNaoKang.

At the end of the treatment, in the WNK group, the degree of improvement in patients’ overall health compared to 1 year ago reflected by Item 2 of the SF‐36 correlated significantly with the improvement in the cognitive function scores recorded by ADAS‐cog (Pearson correlation = 0.680, *P* < 0.010).

### 3.6 Single Photon Emission Computed Tomography results

**FIGURE 2:**
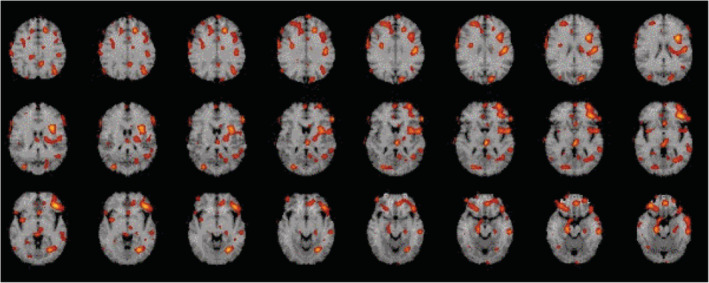
Difference in the changes of cerebral blood flow (t‐scores) pre‐ and post‐treatment between the WNK group (n = 7) and the placebo group (n = 11) Δt > 4.5 (*p* < 0.001). The red color represents the increases in CBF. CBF, cerebral blood flow; WNK, WeiNaoKang.

Among the 18 participants who had post‐treatment scans, 11 were in the placebo group and 7 in the WNK group. The effort required in terms of additional visits and the actual scanning process itself were dis‐incentives to other participants. The 11 paired SPECT scans in the placebo group and the 7 paired SPECT scans in the WNK group, respectively, were standardized by deforming each point in the SPECT scan onto a standard brain MRI using NEUROSTAT and then summed within the group to reduce the noise and the variability of the scans. The statistical results (t‐score data) of each group were displayed on Talairach stereotactic brain space. The difference in change in cerebral blood flow (t‐scores) pre‐ and post‐treatment between the WNK group (n = 7) and the placebo group (n = 11) was statistically significant (*P* < 0.001) and the results are displayed as images (Figure 2).

**FIGURE 3:**
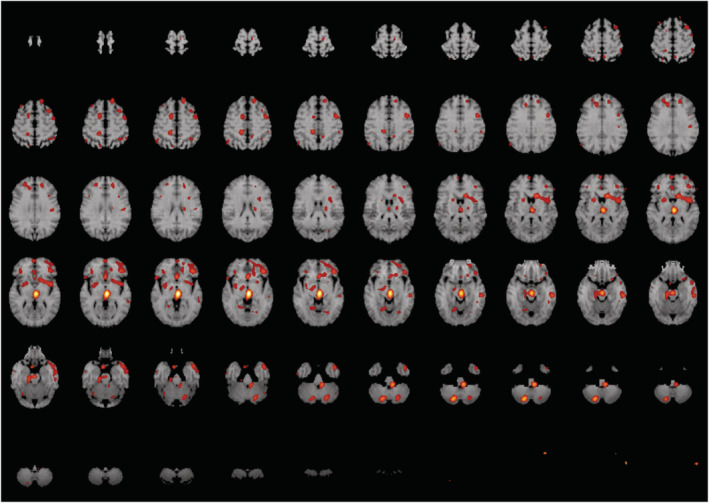
Difference in cerebral blood flow between pre‐ and post‐treatment in the WNK group (*n* = 7), t > 4.5 (*p* < 0.001). The red color represents the increases in CBF. ADAS‐cog, Alzheimer's Disease Assessment Scale – Cognitive Portion; CBF, cerebral blood flow; CI, confidence interval; WNK, WeiNaoKang.

Within the treatment group (n = 7) there appeared to be relatively increased blood flow post‐treatment in the inferior frontal and anterior temporal regions bilaterally, more marked on the left, compared to the pretreatment. The difference was statistically significant *t* > 4.5 (*P* < 0.001) when results were combined and computed as in Figure 3. The apparent increase in the brainstem was seen in both the treatment and the placebo groups and may have been an artifact due to order effect in scanning.

### 3.7 Adverse effects

Two patients withdrew from the trial within the first week of the intervention because of discomfort associated with the treatment. One patient developed sleep disturbance, waking four to five times with difficulty going back to sleep while taking the trial medication. A medical review was conducted by geriatricians and chief investigator on the patient and the decision was made to withdraw the patient from the trial. The second patient withdrew from the trial after gradually developing headache, although similar episodes occurred previously on starting any new medication. The symptom subsided on discontinuation of treatment. Both patients were later found to be in the WNK group.

## 4 DISCUSSION

The pathophysiology of vascular dementia has evolved over time from mere large cerebrovascular diseases to include small blood vessel disease in the subcortical white matter of the brain. The significance of white matter hyperintensities (MRI), especially if extensive and confluent, has attracted much attention in recent years and other mechanisms of pathogenesis for small blood vessels causing cognitive impairment or dementia have been suggested. The most popular hypothesis is that of the breakdown of blood brain barrier due to stroke or ischemia, causing neuro‐inflammation that subsequently results in damage to neuro‐circuits, resulting in cognitive impairment or dementia.^21^, ^22^ This hypothesis has gained further support in recent research that focused on blood brain barrier integrity and dementia,^23^, ^24^ as well as macrophage related chemokines that might be associated with neuro‐inflammation of small vessels of white matter of the brain, resulting in cognitive impairment or VaD.^25^

The WNK (or SLT) consists of three main constituents, ginseng, ginkgo, and saffron, which all have anti‐inflammatory properties, whereas the latter two also have antiplatelet activities. These intrinsic properties of the constituents may be beneficial in the treatment of VaD. Unlike acetylcholinesterase inhibitors (used to treat AD), the main constituents of WNK (or SLT) have intrinsic properties that cover the purported vascular pathology (that is antiplatelet and anti‐inflammatory) that causes VaD, which often co‐exists with AD.^26^, ^27^ This may be the reason why some patients who have been on acetylcholinesterase inhibitor without further cognitive enhancement might have benefited further from the addition of this mixed herb in our study.

The present study showed that WNK is effective in the treatment of cognitive impairment related to VaD and also in improving the patients’ quality of life. Patients receiving the WNK formulation demonstrated significant improvements of the ADAS‐cog and the SF‐36 Health Survey scores than patients receiving the placebo.

Importantly, WNK showed improvement in cerebral blood flow as detected by ^99m^Tc‐HMPAO SPECT. Blood flow was significantly increased in the inferior frontal and anterior temporal regions bilaterally in patients receiving WNK when compared to those from the placebo group (*P* < 0.001). This observation has provided good evidence to support the mechanism by which WNK may improve cognitive function and is consistent with the primary outcome of ADAS‐cog findings.

Our study is the first of its kind to include SPECT that demonstrated an improvement in perfusion to relevant cerebral areas responsible for short term memory. This underpins the possible mechanism at work by this mixed herbal medication. This is also a strength of our design that distinguishes itself from the published SLT study.^5^

Unlike Jia et al's study that consisted of 325 participants with higher doses and longer duration (26 weeks), our current RCT was limited by its smaller sample size (n = 62) and shorter duration (16 weeks). Despite the differences, both RCTs showed statistically significant improvements in cognitive measures that are worthy of note and may have practical benefits to the patients.

Finally, given that there is currently no licensed treatment for VaD, the second most common dementia, which also often exists concomitantly with AD, this mixed herbal medication may be useful in treating VaD, especially in Asian countries where VaD is more prevalent than the West and in countries where cost of treatment of dementia may be an issue, this medication may also be useful as well.

## AUTHOR CONTRIBUTIONS

Conceptualized and designed the study: Chan. Assessed the participants: Chan and Cordato. Contributed to the writing of the manuscript: Chan, Cordato, Chang, Bensoussan, Lee, Dixson, and Liu. Contributed to the design of the study: Chang and Bensoussan. Contributed to the analysis of data: Chang, Bensoussan, Lee, and Liu. Contributed to the design of the SPECT study: Lee and Dixson. Coordinated the study: Liu.

## FUNDING INFORMATION

We received a grant from Western Sydney University and Dr Junguang Liu received a PhD scholarship also from Western Sydney University.

## CONFLICT OF INTEREST

This study was funded by a research grant from Western Sydney University and Dr Junguang Liu received a PhD scholarship. Further studies on Sailuotong are supported by external funding, which is ongoing, but not contributory to the submitted manuscript.

## ETHICS STATEMENT

The trial was conducted in full compliance with the ICH guidelines on Good Clinical Practice (GCP) and the Australian Therapeutic Goods Administration (TGA) guidelines for clinical trials. It was approved by both the Western Sydney University Human Research Ethics Committee (approval no: 04/061) and Sydney South Western Area Health Service Human Ethics Committee (approval no: 04/057).
